# Seeing Inequalities: Self-Reported Visual Difficulty and Eye Care Utilization Among Women in Malawi, a Secondary Analysis of the 2024 Demographic and Health Survey Data

**DOI:** 10.3390/vision10030053

**Published:** 2026-08-14

**Authors:** Selassie Tagoh, Eugene Buah Enimah, Obed Sarpong Adusei, Susarah Maria Richter, Michael Agyemang Kwarteng

**Affiliations:** 1 Department of Medicine, Dunedin School of Medicine, University of Otago, Dunedin 9054, New Zealand; selassie.tagoh@otago.ac.nz; 2 Save Sight Institute, The University of Sydney, Sydney, NSW 2000, Australia; 3 Department of Optometry, Faculty of Science, Bindura University of Science Education, Bindura Private Bag 1020, Zimbabwe; 4 Institute of Public Health and Clinical Nutrition, University of Eastern Finland, FI-70210 Kuopio, Finland; enimah.eugene@gmail.com; 5 Family Medicine Unit, Obuasi Seventh-Day Adventist Hospital, Obuasi BH/99/A, Ghana; yawsarp11@gmail.com; 6 Department of Optometry, Faculty of Health Sciences, University of Johannesburg, Johannesburg 2006, South Africa; 7 Centre for Vision Across the Life Span, Department of Optometry and Vision Science, School of Applied Sciences, University of Huddersfield, Huddersfield HD1 3DH, UK

**Keywords:** visual impairment, reproductive age, visual difficulty, Malawi, spectacle use

## Abstract

Background: This study aimed to identify the factors associated with self-reported visual difficulty and uptake of spectacle/contact lens correction among women of reproductive age in Malawi. Methods: We conducted a cross-sectional secondary analysis of the 2024 Malawi Demographic and Health Survey data. Outcomes were self-reported visual difficulty and use of spectacles/contact lenses. Age, residence, region, education, and marital status were examined as associated variables. Categorical variables were summarized using frequencies and percentages. Chi-square tests, multinomial and binary logistic regression models were used to assess associations. Results: The analysis included 5634 women aged 15–49 years; 78.3% lived in rural areas, and 62.4% had primary education. Self-reported visual difficulty was significantly associated with region, age, residence, education, and marital status (all *p* ≤ 0.004). Reported use of spectacle/contact lenses was also associated with these factors. Increasing age was associated with higher odds of reporting “some difficulty” seeing (OR 1.07, 95% CI 1.06–1.09). Compared with women in the Southern region, those from the Northern (OR 2.1, 95% CI 1.5–2.9) and Central regions (OR 1.6, 95% CI 1.2–2.1) had higher odds of reporting visual difficulty, with stronger associations for “a lot of difficulty” in the Central region (OR 4.3, 95% CI 2.1–8.6). Urban residence was associated with higher odds whereas being married/living together, divorced/separated, or widowed was associated with lower odds. Conclusion: Self-reported visual difficulty and use of spectacles/contact lenses varied by demographic and socioeconomic characteristics. Targeted strategies to improve access to affordable eye care may reduce barriers to uptake of corrective services and reduce visual impairment among women in Malawi.

## 1. Introduction

Aging has a significant effect on the visual system [1]. By early reproductive age, the visual system is fully developed, and any residual refractive error results in significant visual disturbance. Reported risk factors, such as lifestyle, heredity, and the amount of near and outdoor activity, as well as socio-economic status, are known to affect the prevalence of visual disability [2]. In many countries where access to ophthalmic services is underdeveloped, individuals may live with visual impairment for many years [3,4].

The most well-known contributing cause of visual impairment is uncorrected refractive error (URE) [2]. Globally, uncorrected refractive error (URE) is projected to cause visual impairment in 1.8 billion people by 2050 [5]. The associated economic burden of this impairment is estimated at 269 billion dollars annually due to reduced productivity [6].

Visual impairment caused by uncorrected refractive error is preventable by optical corrections when refractive error is the underlying cause [7]. The Vision 2020 initiative contributed significantly to reducing the burden of avoidable visual impairment from uncorrected refractive error and increased global awareness of refractive correction [8,9]. Following these achievements, the current global initiative, 2030 in Sight, now aims to address the inequalities in access and uptake of refractive correction and promote universal access to eye care, especially among women and other vulnerable groups [10].

Visual impairment affects individuals’ quality of life [2]. In children [11], especially learners with disabilities [12,13], it can affect their cognitive, psycho-social and physical development. In adults, good vision is often important for employment and daily livelihoods [2,14]. Among older adults, visual impairment increases the risk of social isolation, depression, falls, and fractures [14,15,16].

Despite the effort to reduce these burdens caused by visual impairment, refractive error remains a health challenge in low-income areas [17,18,19]. Factors such as poverty, lack of insurance coverage for ophthalmic care, educational level and the affordability of correction have been reported to affect the utilization of ophthalmic services [20].

In women of reproductive age, visual difficulty may affect education, employment, caregiving and health service use during this critical life stage. In Malawi, there is limited literature on the uptake of optical correction in general [21,22] and among women of reproductive age.

Visual impairment, URE and self-reported visual difficulty are related aspects, with uncorrected refractive errors contributing to both visual impairment and difficulties in performing visual tasks. In Demographic Health Survey (DHS) questions, what is captured is the perceived difficulty of seeing. Although such surveys do not measure visual acuity to objectively diagnose and determine the cause of visual impairment, they provide information that can be used for identifying inequalities in access to care for specific health conditions, including vision.

In this study, we sought to determine the prevalence of self-reported visual disability among women of reproductive age in Malawi using data from the 2024 Malawi DHS. We also explored factors associated with their uptake of spectacle and contact lens correction.

## 2. Materials and Methods

Malawi is a landlocked country in southern Africa with a predominantly rural population of variable socioeconomic attributes. Its population was approximately 17.5 million at the time of the 2018 census [23] and is projected to reach 22.8 million by 2026. This study analysed data from the 2024 Malawi Demographic Health Survey, conducted nationwide between May and August 2024 [24]. The survey used a stratified cluster-based sampling framework and drew 792 clusters from the 2018 national census [24]. Of the selected households, 22,414 completed the survey, corresponding to a 99% response rate. Visual functioning information was collected from household members of all ages. After excluding respondents with missing vision-related data, 23,095 participants were included in the analysis [22,24]. Detailed information on the survey methodology has been reported by Hanson et al. [22]. This study included data for only women of reproductive age (15–49 years), comprising 5634 respondents.

### 2.1. Study Variables

The main outcome variables were:

1. Self-reported difficulty with vision, classified into five categories: “no difficulty,” “some difficulty,” “a lot of difficulty,” “cannot see at all,” and “don’t know” [24]. These items only capture self-reported visual functioning and do not constitute or distinguish between clinical measures of visual impairment, visual acuity, refractive error or ocular disease.
2. Use of visual correction was defined as one single item (wearing glasses or contact lenses) and scored as “yes” or “no” [24].

These measures were derived from items included in the DHS disability module relating to visual ability. Explanatory variables considered in the analysis included age, sex, geographical region, type of residence (urban or rural), level of education, and marital status.

### 2.2. Data Analysis

Statistical analyses were performed using IBM SPSS Statistics version 31.0.1.0. Categorical variables were summarized using frequencies and percentages. Associations between self-reported visual difficulty, reported use of spectacles/contact lenses, and sociodemographic characteristics were examined using Pearson’s chi-square test. To explore factors associated with levels of visual difficulty, multinomial logistic regression was applied, using “no difficulty seeing” as the reference category. Binary logistic regression was used to identify factors associated with reported use of spectacles/contact lenses. Findings are reported as odds ratios (ORs) with corresponding 95% confidence intervals (CIs), and statistical significance was determined at a threshold of *p* < 0.05.

## 3. Results

### 3.1. Demographic Characteristics of Participants

Data for a total of 5634 women were included in the analysis (Table 1). The age distribution was relatively even across age groups, with the largest proportions in the 30–34, 35–39 and 40–44-year ranges (each ~17%). Most participants resided in the Southern region (53.3%) and in rural areas (78.3%). The majority had attained primary education (62.4%), while only 4.5% had higher education. Most women were either married or living together (43.9%), followed by those who were divorced or separated (40.1%). Among women with available Body Mass Index (BMI) data (*n* = 2826), most had a healthy BMI (63.4%), while 21.8% were overweight and 9.8% were obese.

### 3.2. Distribution of Visual Difficulty According to Demographic Characteristics of Participants

Self-reported visual difficulty varied significantly across demographic groups (Table 2). The distribution of self-reported visual difficulty varied by age, with higher levels reported among women aged 40–49 years (χ^2^ = 212.7, *p* < 0.001). Regional differences were also significant (χ^2^ = 76.3, *p* < 0.001); the southern region had the highest proportion of women reporting severe visual difficulty and responding “don’t know”. Women living in rural areas reported higher levels of visual difficulty than urban residents (χ^2^ = 15.7, *p* = 0.004). Educational attainment was significantly associated with self-reported visual difficulty (χ^2^ = 59.7, *p* < 0.001), with higher reports of difficulty observed among those with lower education levels. Marital status was also associated with self-reported visual difficulty (χ^2^ = 55.3, *p* < 0.001). Specifically, widowed and divorced/separated women reported higher proportions of reported difficulty. In contrast, body mass index was not significantly associated with reported visual difficulty (χ^2^ = 13.9, *p* = 0.124).

### 3.3. Distribution of Glasses/Contact Lens Wear According to Demographic Characteristics and Report of Visual Difficulty

Reported use of glasses or contact lenses varied significantly across demographic characteristics and levels of self-reported visual difficulty (Table 3). Glasses/contact lens use was more common in older women, particularly those aged 40–49 years (χ^2^ = 212.7, *p* < 0.001). Among women who reported “some difficulty” seeing, 23.8% reported using spectacles/contact lenses. The corresponding proportion was 28.65 among those who reported “a lot of difficulty”. Reported use also varied significantly by region (χ^2^ = 76.2, *p* < 0.001), with the highest proportion observed in the Southern region among women reporting “a lot of difficulty” seeing (34.9%). There was a significant association between residence and glasses/contact lens use (χ^2^ = 15.7, *p* = 0.004), with relatively higher proportions reported among rural women, particularly within the “no visual difficulty” (58.3%) and “some difficulty” (54.0%) categories. Educational attainment was also strongly associated with reported glasses/contact lens use (χ^2^ = 59.7, *p* < 0.001), with higher use among those with secondary and higher education. Specifically, 42% of those who reported having some visual difficulty had primary education. Marital status was also significantly associated (χ^2^ = 55.3, *p* < 0.001); 41.3% of those who reported some difficulty and wear glasses/contact lenses are married/living together, while no significant association was observed between spectacle/contact lens use and BMI (χ^2^ = 13.9, *p* = 0.124).

### 3.4. Regression Analysis Showing Demographics According to Visual Difficulty

Using “no difficulty seeing” as the reference, the Multinomial logistic regression model was significant (likelihood ratio χ^2^ = 303.4, df = 28, *p* < 0.001) and the goodness of fit test did not indicate lack of fit (χ^2^ = 1988.8, df = 2472, *p* = 1.00) (Table 4). Increasing age was associated with higher odds of reporting “some difficulty” with seeing (OR 1.07, 95% CI 1.06–1.09, *p* < 0.001) and a “don’t know” response (OR: 1.09, 95% CI 1.07–1.10, *p* < 0.001) but was not significantly associated with “cannot see at all.” Compared with women in the Southern region, women in the Northern (OR 2.1, 95% CI 1.5–2.9, *p* < 0.001) and Central regions (OR: 1.6, 95% CI 1.2–2.1, *p* = 0.003) had higher odds of reporting “some difficulty” seeing. Women in the central region also had higher odds of reporting “a lot of difficulty” (OR: 4.3, 95% CI 2.1–8.6, *p* < 0.001) in seeing. Urban residence was associated with 40% odds of reporting “some difficulty” (OR: 1.4, 95% CI 1.1–1.9, *p* = 0.020). Compared with the reference marital-status group, women who were married/living with a partner (OR: 0.3, 95% CI 0.2–0.5, *p* < 0.001), divorced/separated (OR: 0.3, 95% CI 0.2–0.5, *p* < 0.001) or widowed (OR: 0.3, 95% CI 0.1–0.5, *p* < 0.001) had lower odds of reporting “some difficulty”. BMI showed no consistent association with self-reported visual difficulty. Estimates for the “cannot see at all” category were unstable due to sparse data and are likely unreliable.

### 3.5. Binary Logistic Regression Showing Factors Associated with Reports of Glasses or Contact Lens Wear

In the binary logistic regression analysis (Table 5), increasing age was associated with slightly higher odds of reported use of glasses or contact lenses (OR: 1.06, 95% CI 1.04–1.08, *p* < 0.001). Education showed a strong association, with participants having primary (OR: 2.7, 95% CI 1.5–5.1, *p* = 0.002) and especially secondary education (OR: 10.7, 95% CI 5.4–21.2, *p* < 0.001) demonstrating significantly higher odds of reported glasses/contact lens use compared to the reference group. Overweight individuals also had higher odds of reported glasses/contact lens use (OR: 3.9, 95% CI 1.3–11.3, *p* = 0.013). Urban residence was associated with lower odds of reported glasses/contact lens use (OR: 0.7, 95% CI 0.5–1.0, *p* = 0.049). Region, marital status, and most BMI categories were not significantly associated with the self-reported use of glasses/contact lenses.

### 3.6. District-Level Distribution of Self-Reported Visual Difficulty and Glasses/Contact Lens Use

**Figure 1:**
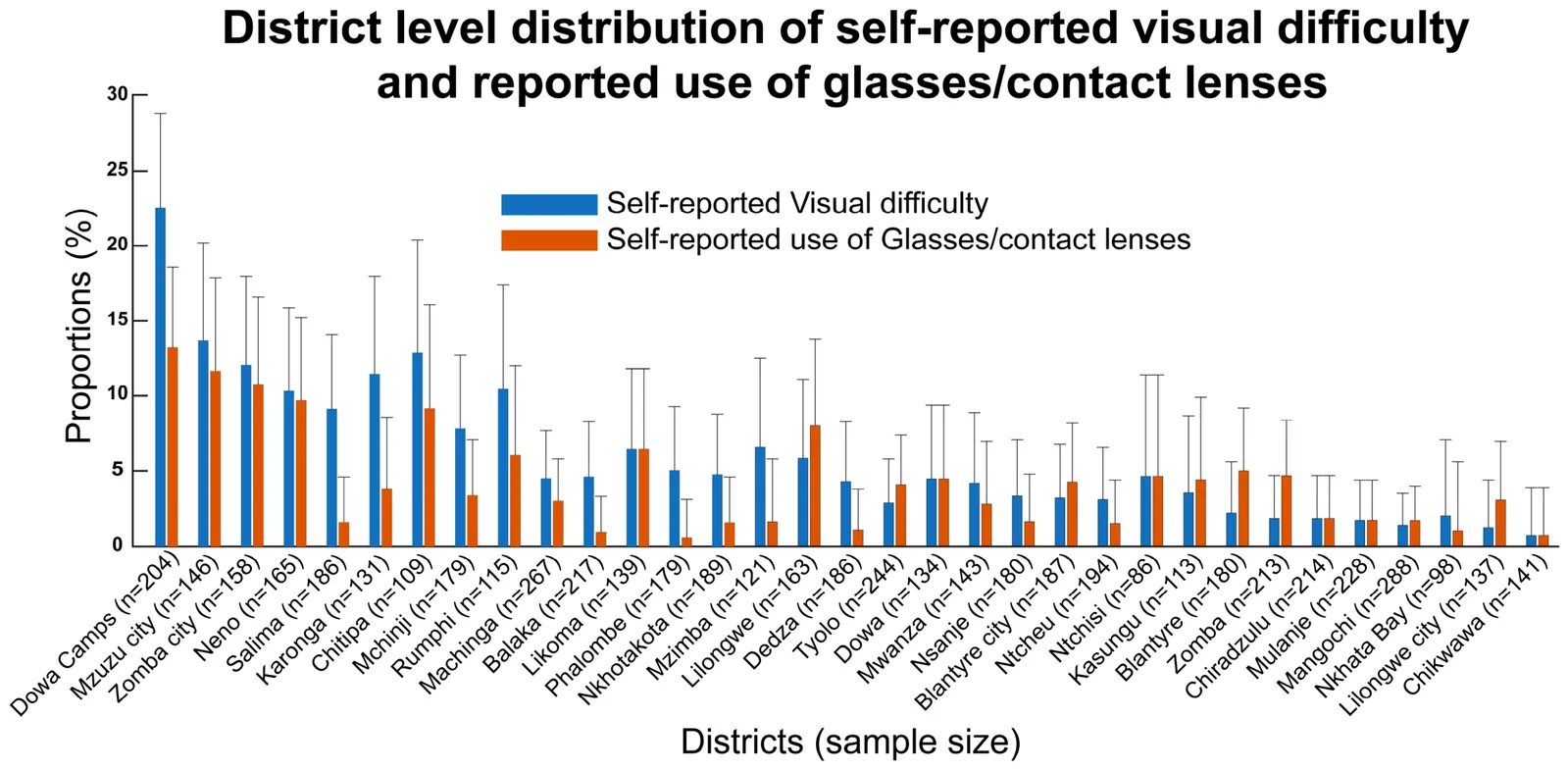
Proportion of self-reported visual difficulty and reported use of glasses/contact lenses among women aged 15–49 years across districts in Malawi. Bars represent the proportion of women in each district reporting visual difficulty (blue) and wearing glasses or contact lenses (Red). Error bars represent 95% Confidence intervals. Visual difficulty was defined as reporting any level of difficulty seeing (“some difficulty”, “a lot of difficulty”, or “unable to see”). Substantial variation is observed across districts, with a high proportion in Dowa (camps), where 22% of women reported having visual difficulty but only 13% reported wearing glasses or contact lenses. The lowest rate was in Chikwawa, where the only woman who reported having visual difficulty also reported wearing glasses/contact lenses.

There was substantial variation in the proportions of self-reported visual difficulty across districts, ranging from approximately 0.7% in Chikwawa to 22.5% in Dowa (camps) (Figure 1). Relatively higher percentages were also observed in Mzuzu City (13.7%) and Zomba City (12.0%), whereas Mangochi (1.4%), Zomba (1.9%), and Chiradzulu (1.9%) had lower proportions. Reported use of glasses or contact lenses likewise varied by districts, from 0.6% in Phalombe to 13.2% in Dowa (camps), with medium levels observed in Mzuzu City (11.6%) and Zomba City (10.8%). The district-level patterns of self-reported visual difficulty and glasses/contact lens use did not correspond consistently. For example, Blantyre (2.2% visual difficulty vs. 5.0% glasses use) and Zomba (1.9% vs. 4.7%) had relatively higher reported glasses/contact lens use despite low self-reported visual difficulty, whereas most of the other regions, including Phalombe (5.0% vs. 0.6%), showed the reverse pattern.

## 4. Discussion

This study examined the demographic, socioeconomic, and geographic factors associated with self-reported visual difficulty and reported use of glasses and contact lenses among women of reproductive age in Malawi. The findings revealed variation in both outcomes across population groups and districts. While the pattern of results reported here does not provide objective evidence of measured visual impairment, refractive error or direct access to care, they reveal persistent inequalities in eye health across populations. Such disparities are consistent with global evidence indicating that vision impairment remains a major public health issue, particularly in low- and middle-income countries where access to eye care services is limited [25].

Most women in the study population lived predominantly in rural areas, resided in the Southern region, and had primary education. This reflects broader patterns in sub-Saharan Africa, where structural inequalities such as lower educational attainment and rural residence significantly influence health outcomes and access to care [20]. These contextual factors are important determinants of both visual health status and healthcare utilisation [20].

Increasing age was associated with higher odds of self-reported visual difficulty, particularly among women aged 40–49 years. This pattern is consistent with evidence that vision problems become more common with age [26,27,28], largely due to conditions such as presbyopia, cataracts, and other age-related ocular diseases. Indeed, presbyopia alone affects over one billion people globally, with a disproportionately high unmet need in low-resource settings [26,27,28]. These findings reinforce the need for age-targeted vision screening and intervention programmes in vulnerable population groups.

Geographic and residential disparities were also evident in the results, with the regression analysis results suggesting higher odds of self-reported visual difficulty among women in the Northern and Central regions and among urban residents. These associations may reflect differences in the composition of the population or unmeasured social, economic and healthcare-related factors such as differences in age structure, socioeconomic status, or healthcare access. Evidence from Malawi and other sub-Saharan African countries indicates that the distribution and accessibility of eye care services can be shaped by geographical and systemic factors, including workforce shortages, location of services (urban concentration of services), and transportation barriers [19,20,29,30]. These findings highlight the importance of multivariate approaches in disentangling healthcare disparities.

Educational attainment was associated with both self-reported visual difficulty and reported use of glasses or contact lenses. Women with lower educational attainment were more likely to report visual difficulty, while those with primary and secondary education had significantly higher odds of reporting use of glasses or contact lenses. This pattern is broadly consistent with literature linking educational attainment to visual health outcomes and use of glasses, contributing to greater prevalence of visual impairment [31]. Moreover, educational interventions have been shown to increase knowledge of vision problems and significantly improve uptake of spectacles, particularly when combined with access initiatives. This was the case in rural China, where vision health education combined with subsidized eyeglasses initiatives led to notable gains in both eye health knowledge and spectacle use [32]. A broader review of school-based eye health programmes in low- and middle-income countries found that education and free spectacle provision were most effective at enhancing spectacle compliance and improving knowledge, attitudes, and practices [33]. These findings suggest that education may influence how respondents understand and report visual difficulty, which may affect their likelihood of receiving appropriate care.

Marital status was associated with self-reported visual difficulty in the adjusted analysis. Women who were widowed or divorced/separated had lower odds of reporting “some difficulty” seeing than women in the reference marital status group. This finding was unexpected, as widowhood and marital disruptions are often associated with poorer health outcomes. Although a multi-country population-based study of adults aged 18–49 years in Ghana, Gambia and Togo found that marital status remained significantly associated with self-reported visual difficulties even after adjusting for sociodemographic factors [34], we note that the relationship between marital status and self-reported visual difficulty may be context-specific and influenced by individual, social, cultural or healthcare related factors [22,35], that were not fully captured in this study. Hence, the unexpectedly lower odds associated with widowed and divorced/separated women may reflect residual confounding by these unmeasured factors.

BMI was not significantly associated with self-reported visual difficulty. Reported use of glasses/contact lenses was higher among women classified as overweight, whereas the other BMI categories showed no significant associations. This pattern is consistent with prior work showing weak links between BMI and visual impairment in similar populations [36].

Overall, reported use of glasses/contact lenses reflected visual needs and varied by age, education, residence and district. Older women and women reporting greater visual difficulty were more likely to report using glasses or contact lenses. There was also higher reported use in rural areas but lower odds of reported use in urban areas after adjustment. Such discrepancies may relate to variations in factors such as awareness, affordability, cultural acceptance, previous eye examination and perceived individual needs, rather than access alone [37]. Education remained a strong factor influencing the reported use of glasses or contact lenses, reinforcing evidence that greater health literacy and exposure to information improve uptake of vision correction [4]. District-level analysis revealed substantial geographic variation in visual difficulty and self-reported use of glasses/contact lenses, reflecting resource disparities and service delivery gaps documented across Africa, and highlighting the need for locally tailored interventions [7,17,18,38].

Several limitations should be considered when interpreting these findings. First, the study relied on self-reported visual difficulty, which is a subjective indicator of perceived visual functioning and may introduce reporting bias, as subjective assessments do not always align with objective clinical measures of visual impairment. Studies have shown that self-reported vision can both overestimate and underestimate true visual impairment depending on individual and contextual factors [39,40]. Second, the data analysed lacked information on household wealth index, occupation, health insurance, eye-care accessibility, previous eye examinations, appropriateness of glasses being worn and chronic systemic diseases with ocular manifestations like diabetes and hypertension. These unmeasured factors may have confounded the observed associations, particularly with BMI. Third, the DHS item combined glasses and contact lens use; hence, the study is unable to estimate the use of each modality separately. Finally, the cross-sectional design of the study limits causal inference.

## 5. Conclusions

This study shows that self-reported visual difficulty and the reported use of spectacles or contact lenses among women in Malawi are influenced by a complex interplay of age, education, geographic location, and other social determinants. Older and less-educated women appear particularly vulnerable. Reported access to corrective lenses is strongly influenced by educational attainment and varies significantly across regions and districts. Given that a large proportion of visual difficulty is preventable or treatable with relatively simple interventions such as glasses or contact lenses, information on self-reported visual difficulty and use of spectacles will guide public health policies aimed at improving access to affordable eye care and eye health education, particularly in underserved populations. Targeted strategies, including community-based screening, health education, and expansion of subsidised spectacle provision initiatives, are essential to reduce inequalities and address the unmet need for vision care among women of reproductive age and other vulnerable groups.

## Figures and Tables

**Table 1:** Demographic characteristics of participants.

Variables	Categories	Frequency (Percentage)
Age (years)	15–19	207 (3.7)
	20–24	855 (15.2
	25–29	938 (16.6)
	30–34	971 (17.2)
	35–39	969 (17.2)
	40–44	967 (17.2)
	45–49	727 (12.9)
Sex	Females	5634 (100)
Region	Northern	859 (15.2)
	Central	1771 (31.4)
	Southern	3004 (53.3)
Type of residence	Urban	1221 (21.7)
	Rural	4413 (78.3)
Highest education level attained	No education, preschool/early childhood	543 (9.6)
	Primary	3513 (62.4)
	Secondary	1317 (23.4)
	Higher	252 (4.5)
	Don’t know	9 (0.2)
Marital status	Married or living together	2475 (43.9)
	Divorced/separated	2260 (40.1)
	Widowed	523 (9.3)
	Never married or lived together	376 (6.7)
* Body Mass Index(kg/m^2^)	Underweight (≤18.5)	142 (5.0)
	Healthy Weight (18.6–24.9)	1793 (63.4)
	Overweight (24.91–29.9)	615 (21.8)
	Obese (≥30.0)	276 (9.8)

* Out of the total number of 5634 women, BMI data was available for only 2826.

**Table 2:** Distribution of self-reported visual difficulty according to demographic characteristics of participants.

Variables	Categories	No Visual Difficulty (%)	Some Difficulty	A Lot of Difficulty	Can’t See at All	Don’t Know	Pearson Chi-Square (χ^2^)	*p*-Value
Age	15–19	201 (4.0)	4 (1.5)	0 (0.0)	0 (0.0)	2 (0.8)	212.7	**<0.001**
	20–24	809 (15.9)	26 (9.8)	5 (9.8)	0 (0.0)	15 (6.4)		
	25–29	881 (17.3)	30 (11.4)	6 (11.8)	0 (0.0)	21 (8.9)		
	30–34	900 (17.7)	32 (12.1)	8 (15.3)	0 (0.0)	31 (13.1)		
	35–39	896 (17.6)	35 (13.3)	5 (9.8)	0 (0.0)	33 (14.0)		
	40–44	829 (16.3)	63 (23.9)	12 (23.5)	1 (50.0)	62 (26.3)		
	45–49	565 (11.1)	74 (28.0)	15 (29.4)	1 (50.0)	72 (30.5)		
Sex	Females	5081 (100)	264 (100)	51 (100)	2 (1000	236 (100)	-	-
Region	Northern	739 (14.5)	69 (26.1)	11 (21.6)	0 (0.0)	40 (16.9)	76.3	**<0.001**
	Central	1602 (31.5)	95 (36.0)	29 (56.9)	0 (0.0)	459 (19.1)		
	Southern	2740 (53.9)	100 (37.9)	11 (21.6)	2 (100)	151 (64.0)		
Type of residence	Urban	1070 (21.1)	80 (30.3)	15 (29.4)	0 (0.0)	56 (23.7)	15.7	**0.004**
	Rural	4011 (78.9)	184 (69.7)	36 (70.6)	2 (100)	180 (76.3)		
Highest education level	No education	483 (9.50	24 (9.1)	6 (11.8)	0 (0.0)	30 (12.7)	59.7	**<0.001**
	Primary	3189 (62.8)	153 (58.0)	23 (45.1)	2 (100)	146 (61.9)		
	Secondary	1198 (23.6)	53 (20.1)	18 (35.3)	0 (0.0	48 (20.3)		
	Higher	202 (4.0)	34 (12.9)	4 (7.8)	0 (0.0)	12 (5.1)		
	Don’t know	9 (0.2)	0 (0.0)	0	0 (0.0)	0 (0.0)		
Marital status	Married or living together	2289 (45.1)	98 (37.1)	14 (27.5)	0 (0.0)	74 (31.4)	55.3	**<0.001**
	Divorced/separated	2022 (39.8)	106 (40.2)	22 (43.1)	1 (50)	109 (46.2)		
	Widowed	443 (8.7)	30 (11.4)	11 (21.6)	1 (50)	38 (16.1)		
	Never married or lived together	327 (6.4)	30 (11.4)	4 (7.8)	0 (0.0)	15 (6.4)		
* Body Mass Index(kg/m^2^)	Underweight (≤18.5)	128 (5.1)	6 (4.1)	0 (0.0)	0 (0.0)	8 (6.5)	13.9	0.124
	Healthy Weight (18.6–24.9)	1622 (64.1)	79 (53.7)	13 (52.0)	0 (0.0)	79 (63.7)		
	Overweight (24.91–29.9)	538 (21.3)	41 (27.9)	8 (32.0)	0 (0.0)	28 (22.6)		
	Obese (≥30.0)	242 (9.6)	21 (14.3)	4 (16.0)	0 (0.0)	9 (7.3)		

Note: * Out of the total number of 5634 women, BMI data was available for only 2826. Bold *p*-value represents significant association.

**Table 3:** Distribution of glasses/contact lens wear according to demographic characteristics and report of visual difficulty.

Variables	Categories	No Visual Difficulty (%)		Some Difficulty		A Lot of Difficulty		Can’t See at All		Don’t Know		Pearson Chi-Square (χ^2^)	*p*-Value
		Wear Glasses/Contact Lenses		Wear Glasses/Contact Lenses		Wear Glasses/Contact Lenses		Wear Glasses/Contact Lenses		Wear Glasses/Contact Lenses			
		Yes (%)	No (%)	Yes (%)	No (%)	Yes (%)	No (%)	Yes (%)	No (%)	Yes (%)	No (%)		
Age	15–19	4 (3.0)	197 (4%)	0 (0.0)	4 (2.0)	0 (0.0)	0 (0.0)	0 (0.0)	0 (0.0)	1 (9.1)	1 (0.4)	212.7	**<0.001**
	20–24	8 (6.1)	801 (16.2)	8 (12.7)	18 (9.0)	4 (19.0)	1 (3.3)	0 (0.0)	0 (0.0)	0 (0.0)	15 (6.7)		
	25–29	19 (14.4)	862 (17.4)	9 (14.3)	21 (10.4)	1 (4.8)	5 (16.7)	0 (0.0)	0 (0.0)	1 (9.1)	20 (8.9)		
	30–34	22 (16.7)	878 (17.7)	11 (17.5)	21 (10.4)	4 (19.0)	4 (13.3)	0 (0.0)	0 (0.0)	2 (18.2)	29 (12.9)		
	35–39	21 (15.9)	875 (17.7)	5 (7.9)	30 (14.9)	3 (14.3)	2 (6.7)	0 (0.0)	0 (0.0)	2 (18.2)	31 (13.8)		
	40–44	31 (23.5)	798 (16.1)	15 (23.8)	48 (23.9)	3 (14.3)	9 (30.0)	0 (0.0)	1 (100)	2 (18.2)	60 (26.7)		
	45–49	27 (20.5)	538 (10.9)	15 (23.8)	59 (29.4)	6 (28.6)	9 (30.0)	1 (100)	0 (0.0)	3 (27.3)	69 (30.7)		
Region	Northern	26 (19.7)	713 (14.4)	19 (30.2)	50 (24.9)	5 (23.8)	6 (20.0)	0 (0.0)	0 (0.0)	1 (9.1)	39 (17.3)	76.2	**<0.001**
	Central	38 (28.8)	1564 (31.6)	22 (34.9)	73 (36.3)	12 (57.1)	17 (56.7)	0 (0.0)	0 (0.0)	3 (27.3)	42 (18.7)		
	Southern	68 (51.5)	2672 (54.0)	22 (34.9)	78 (38.8)	4 (19.0)	7 (23.3)	1 (100)	1 (100)	7 (63.6)	144 (64.0)		
Residence	Urban	55 (41.7)	1015 (20.5)	29 (46.0)	51 (25.4)	4 (19.0)	11 (36.7)	0 (0.0)	0 (0.0)	3 (27.3)	53 (23.6)	15.7	**0.004**
	Rural	77 (58.3)	3934 (79.5)	34 (54.0)	150 (74.6)	17 (81.0)	19 (63.3)	1 (100)	1 (100)	8 (72.7)	172 (76.4)		
Education level	No education	8 (6.1)	475 (9.6)	4 (6.3)	20 (10.0)	1 (4.8)	5 (16.7)	0 (0.0)	0 (0.0)	0 (0.0)	30 (13.3)	59.7	**<0.001**
	Primary	49 (37.1)	3140 (63.4)	25 (39.7)	128 (63.7)	11 (52.4)	12 (40.0)	1 (100)	1 (100)	4 (36.4)	142 (63.1)		
	Secondary	49 (37.1)	1149 (23.2)	11 (17.5)	42 (20.9)	7 (33.3)	11 (36.7)	0 (0.0)	0 (0.0)	5 (45.5)	43 (19.1)		
	Higher	25 (18.9	177 (3.6)	23 (36.5)	11 (5.5)	2 (9.5)	2 (6.7)	0 (0.0)	0 (0.0)	2 (18.2)	10 (4.4)		
	Don’t know	1 (0.8)	8 (0.2)	0 (0.0)	0 (0.0)	0 (0.0)	0 (0.0)	0 (0.0)	0 (0.0)	0 (0.0)	0 (0.0)		
Marital status	Married or living together	60 (45.5)	2229 (45.0)	26 (41.3)	72 (35.8)	7 (33.3)	7 (23.3)	0 (0.0)	0 (0.0)	4 (36.4)	70 (31.1)	55.3	**<0.001**
	Divorced/separated	44 (33.3)	1978 (40.0)	19 (30.2)	87 (43.3)	9 (42.9)	13 (43.3)	0 (0.0)	1 (100)	3 (27.3)	106 (47.1)		
	Widowed	15 (11.4)	428 (8.6)	6 (9.5)	24 (11.9)	3 (14.3)	8 (26.7)	1 (100)	0 (0.0)	1 (9.1)	37 (16.4)		
	Never married or lived together	13 (9.8)	314 (6.3)	12 (19.0)	18 (9.0)	2 (9.5)	2 (6.7)	0 (0.0)	0 (0.0)	3 (27.3)	12 (5.3)		
* Body Mass Index(kg/m^2^)	Underweight (≤18.5)	2 (2.5)	126 (5.1)	2 (5.3)	4 (3.7)	0 (0.0)	0 (0.0)	0 (0.0)	0 (0.0)	0 (0.0)	8 (6.8)	13.9	0.124
	Healthy Weight (18.6–24.9)	36 (45.6)	1586 (64.7)	14 (36.8)	65 (59.6)	6 (50.0)	7 (53.8)	0 (0.0)	0 (0.0)	5 (83.3)	74 (62.7)		
	Overweight (24.91.0–29.9)	24 (30.4)	514 (21.0)	14 (36.8)	27 (24.8)	4 (33.3)	4 (30.8)	0 (0.0)	0 (0.0)	0 (0.0)	28 (23.7)		
	Obese (≥30.0)	17 (21.5)	225 (9.2)	8 (21.1)	13 (11.9)	2 (16.7)	2 (15.4)	0 (0.0)	0 (0.0)	1 (16.7)	8 (6.8)		

Note: * Out of the total number of 5634 women, BMI data was available for only 2826. Bold *p*-value represents significant association.

**Table 4:** Regression analysis showing demographics according to visual difficulty.

Predictors	Some Difficulty OR (95% CI)	*p*-Value	A Lot of Difficulty OR (95% CI)	*p*-Value	Cannot See at All OR (95% CI)	*p*-Value	Don’t Know OR (95% CI)	*p*-Value
Age	1.07 (1.06–1.09	**<0.001**	1.06 (1.02–1.1)	**0.002**	1.2 (0.9–1.6)	0.249	1.09 (1.07–1.1)	**<** **0** **.001**
Northern Region	2.1 (1.5–2.9)	**<0.001**	3.0 (1.3–7.1)	**0.011**	* 1.3 × 10^−7^	0.997	0.8 (0.6–1.2)	0.271
Central Region	1.6 (1.2–2.1)	**0.003**	4.3 (2.1–8.6)	**<0.001**	* 9.9 × 10^−8^	0.995	0.5 (0.3–0.7)	**<** **0** **.001**
Urban Residence	1.4 (1.1–1.9)	**0.020**	1.4 (0.8–2.7)	0.258	2.7 × 10^−7^	0.996	1.1 (0.8–1.5)	0.512
Married/Living together	0.3 (0.2–0.5)	**<0.001**	0.34 (0.1–1.1)	0.081	* 0.145	1.000	0.3 (0.2–0.6)	**<** **0** **.001**
Divorced/separated	0.3 (0.2–0.5)	**<0.001**	0.6 (0.2–1.8)	0.330	* 3.3 × 10^6^ (1.8 × 10^5^–6.1 × 10^7^)	**<** **0** **.001**	0.5 (0.3–0.9)	**0** **.019**
Widowed	0.3 (0.1–0.5)	**<0.001**	0.9 (0.2–3.1)	0.816	* 8.6 × 10^6^	-	0.5 (0.3–1.0)	**0** **.046**
Underweight (≤18.5)	0.5 (0.2–1.4)	0.195	* 3.3 × 10^−9^	-	-	-	1.7 (0.6–4.4)	0.297
Healthy Weight (18.6–24.9)	0.6 (0.3–0.9)	**0.024**	0.5 (0.2–1.5)	0.209	-	-	1.3 (0.6–2.6)	0.452
Overweight (24.91.0–29.9)	0.9 (0.5–1.5)	0.642	0.9 (0.3–3.0)	0.864	-	-	1.4 (0.7–3.0)	0.390

* ORs were extremely large or small with extremely wide or undefined 95% CIs; these estimates are likely unreliable. Sex was also excluded because it has only 1 category (Females). The estimates for the level of education were unreliable and thus excluded. Collapsing the cells with small numbers into one may lead to loss of information, as each visual difficulty category provides unique information, so this was not done. No observations were available for BMI for those who cannot see at all; hence, these cells are empty. Bold *p*-value represents significant association.

**Table 5:** Binary Logistic regression showing the factors associated with use of glasses or contact lens wear.

Predictors	Odds Ratio (OR)	95% Confidence Interval (CI)		*p*-Value
		Lower Bound	Upper Bound	
Age	1.06	1.04	1.08	**<0.001**
Northern Region	0.9	0.6	1.3	0.529
Central Region	0.8	0.5	1.1	0.122
Urban Residence	0.7	0.5	1.0	**0.049**
No Education	1.3	0.7	2.3	0.432
Primary Education	2.7	1.5	5.1	**0.002**
Secondary education	10.7	5.4	21.2	**<0.001**
Higher Education	4.5	0.5	39.1	0.174
Married/Living together	0.8	0.6	1.1	0.163
Divorced/separated	0.9	0.5	1.4	0.541
Widowed	1.2	0.7	2.0	0.442
Underweight (≤18.5)	1.2	0.435	3.391	0.710
Healthy Weight (18.6–24.9)	2.5	0.9	7.2	0.081
Overweight (24.91–29.9)	3.9	1.3	11.3	**0.013**

Bold *p*-value represents significant association.

## Data Availability

Data available on request from the DHS and can be downloaded from the official DHS website: http://www.dhsprogram.com (accessed on 21 April 2026).
